# *TLR4* and *MD2* variation among horses with differential TNFα baseline concentrations and response to intravenous lipopolysaccharide infusion

**DOI:** 10.1038/s41598-023-27956-y

**Published:** 2023-01-27

**Authors:** Abhijit Mukhopadhyay, Shawna R. Cook, Phillip SanMiguel, Kari J. Ekenstedt, Sandra D. Taylor

**Affiliations:** 1 Department of Veterinary Clinical Sciences, College of Veterinary Medicine, Purdue University, West Lafayette, IN USA; 2 Department of Basic Medical Sciences, College of Veterinary Medicine, Purdue University, West Lafayette, IN USA; 3 Bindley Bioscience Center, Purdue University, West Lafayette, IN USA

**Keywords:** Genetics, Immunology, Medical research

## Abstract

Gram-negative bacterial septicemia is mediated through binding of lipopolysaccharide (LPS) to mammalian toll-like receptor protein 4 (TLR4). TLR4 and its cognate protein, myeloid differentiation factor 2 (MD2) form a heterodimeric complex after binding LPS. This complex induces a cascade of reactions that results in increased proinflammatory cytokine gene expression, including TNFα, which leads to activation of innate immunity. In horses, the immune response to LPS varies widely. To determine if this variation is due to differences in TLR4 or MD2, DNA from 15 healthy adult horses with different TNFα dynamics after experimental intravenous LPS infusion was sequenced across exons of *TLR4* and *MD2*. Haplotypes were constructed for both genes using all identified variants. Four haplotypes were observed for each gene. No significant associations were found between either TNFα baseline concentrations or response to LPS and haplotype; however, there was a significant association (*P* value = 0.0460) between the baseline TNFα concentration and one *MD2* missense variant. Three-dimensional structures of the equine TLR4-MD2-LPS complex were built according to haplotype combinations observed in the study horses, and the implications of missense variants on LPS binding were modeled. Although the sample size was small, there was no evidence that variation in TLR4 or MD2 explains the variability in TNFα response observed after LPS exposure in horses.

## Introduction

Despite substantial advances in medical management, gram negative septicemia continues to be a significant cause of morbidity and mortality in horses^1,2^. During gram negative septicemia, lipopolysaccharide binding protein binds the lipid A moiety of lipopolysaccharide (LPS; endotoxin) and transfers LPS to soluble (plasma) CD14 or membrane-bound CD14 on macrophages. This complex then binds and activates the toll-like receptor-4 (TLR4) and myeloid differentiation factor-2 (MD2) complex, which initiates signal transduction to ultimately induce production and release of pro-inflammatory cytokines such as TNFα^3^. Compared to other species, horses are particularly sensitive to LPS^4,5^, the deleterious effects of which are mediated through this TLR4 cell signaling. Given the relative sensitivity of horses to LPS, a non-lethal, low-dose (30 ng/kg) intravenous (IV) LPS administration model of gram negative septicemia has been established that can induce transient and systemic inflammation in the horse^6^. This model has been used extensively to investigate the clinical and clinicopathologic abnormalities associated with equine gram negative septicemia, and to test the efficacy of various drugs^7–9^. However, the individual equine response to IV LPS administration is variable^9–12^. In horses with a strong inflammatory response to low-dose IV LPS infusion, clinical signs such as pyrexia, tachycardia, tachypnea, and colic occur in conjunction with neutropenia and a ≥ 50% increase in TNFα serum concentration within one hour^7,13^. Yet, in a study investigating the effects of ascorbic acid and hydrocortisone as a treatment protocol after infusion of low-dose IV LPS, eight of 40 horses were excluded due to complete lack of clinical and clinicopathological response to LPS^9^. Reports of naturally occurring cases of equine gram negative septicemia also demonstrate this variation in response^14^. To date, factors that predict the clinical, clinicopathological, or cytokine response to LPS have not been identified in the horse.

The variation in response to LPS might be a result of molecular differences in equine *TLR4* and/or *MD2*. While *E. coli* LPS is a TLR4 agonist across all species^15,16^, previous in vitro work has determined that equine TLR4 and MD2 are unique. For example, the TLR4/MD2 complex responds differently to LPS from *Rhodobacter sphaeroides* in equine cells, where it is an LPS agonist, versus human cells, where it is an LPS antagonist^17^. Indeed, discrete regions of both MD2 and TLR4 appear to be required for lipid IVa signaling, based on in vitro studies (lipid IVa being an LPS derivative that is an antagonistic in humans but an agonist in horses)^18^. Conversely, DiC14-amidine nanoliposomes are weak agonists in the horse, but strong agonists in human; here, two TLR4 regions were identified that modulated the human agonist activity of diC14-amidine, but both regions are outside the known LPS-binding domain^15^. Antagonists typically act by binding to monomeric TLR4/MD2 complexes in a non-dimerizing manner, preventing signaling^16^; it is reasonable to hypothesize that activity of antagonists could also be affected by differences in the TLR4 and/or MD2 proteins, even within a species.

Although the intracellular domains of human and murine TLR4 are highly conserved across species, the extracellular domains that contain the LPS binding site exhibit considerable sequence divergence in humans and mice^19,20^. Variations in this extracellular domain can ultimately change receptor affinity and specificity to LPS^4^. Human and equine *TLR4* genes are similar (86.1% similarity [amino acid sequence homology]), comparing human NP_612564.1 and horse NP_001093239.2 in NCBI’s BLAST [https://blast.ncbi.nlm.nih.gov/Blast.cgi] using the Needleman-Wunsch global alignment)^21^; however, divergence between species in the TLR4 extracellular domain is expected^22^. In a small study of five horses, four expressed single nucleotide polymorphisms (SNPs) in the *TLR4* gene but to date, the significance of these are unknown^23^. In fact, the specific LPS binding site on TLR4 has not been defined in the horse, although, as mentioned above, the interactions of the equine TLR4/MD2 complex with various other agonists and antagonists have been defined. As with *TLR4*, the human and equine *MD2* genes are similar (81% similarity, comparing human NP_056179.4 and horse NP_001075367.1 as above)^21^, but sequence variations in *MD2* and the potential effects on LPS response among individual horses is unknown. In humans and mice, the three-dimensional (3D) crystal structure of the TLR4-MD2-LPS complex has been determined^24,25^, and these can be used to predict the equine TLR4-MD2-LPS 3D structure. Sequence differences in *TLR4* and *MD2* resulting in changes to their respective proteins might lead to conformational changes that affect LPS binding and subsequent clinical responses to gram negative infections^19,26^.

The first objective of this study was to sequence the exons of *TLR4* and *MD2* from 15 horses that were administered IV LPS. Amino acid (AA) sequences of both proteins were derived from gene sequencing. For both genes, both individual variants and constructed haplotypes were analyzed for association with baseline TNFα concentration and TNFα response to LPS. A second objective of the study was to describe the LPS binding sites on equine TLR4 and MD2 based on molecular modeling (as observed in the study horses) and determine the extent to which variations in either protein could affect LPS binding.

## Results

### TNFα concentrations at, and correlation between, baseline and response

**Figure 1:**
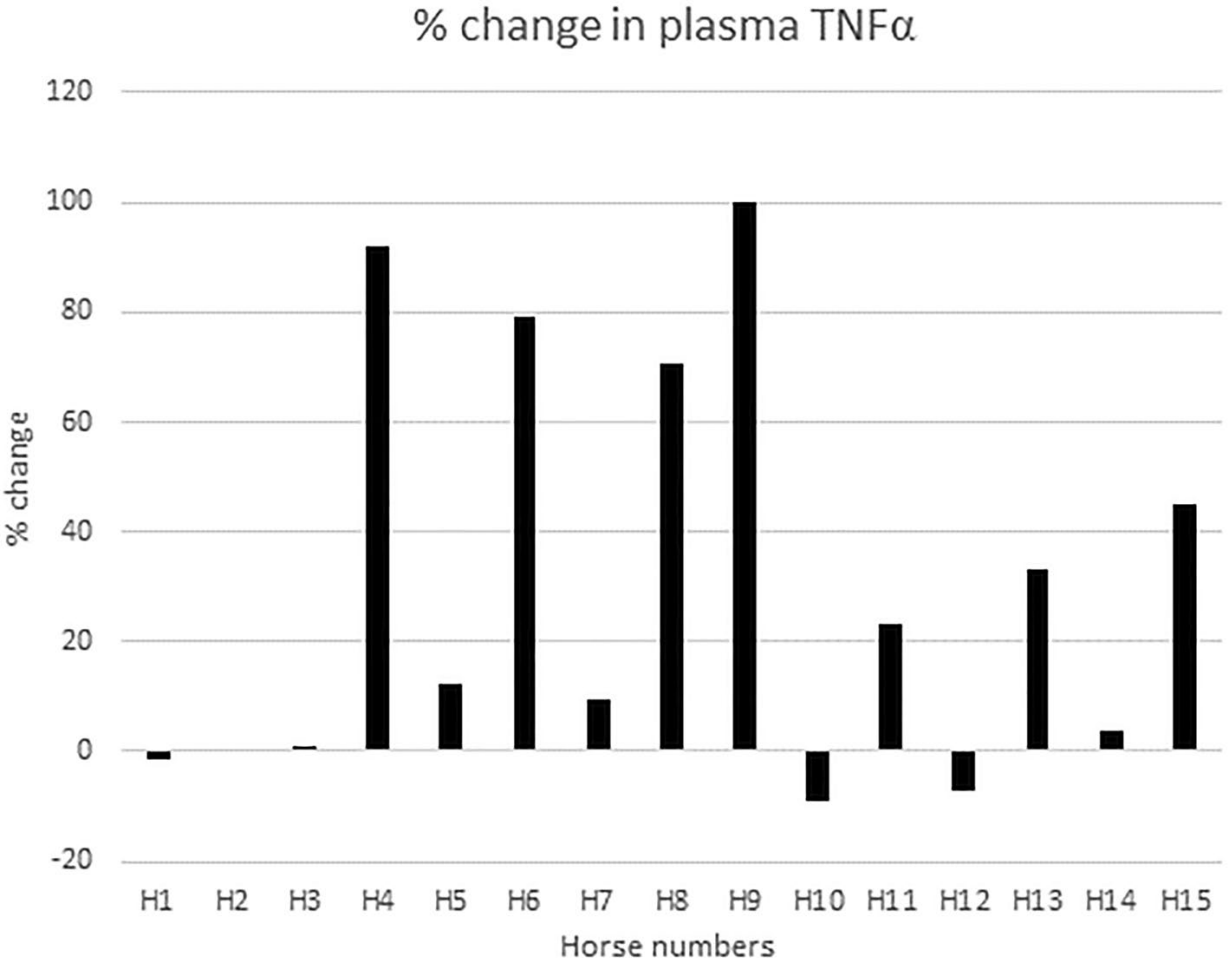
Percent change in plasma TNFα concentration. The percent change in plasma TNFα concentration one hour after IV LPS infusion compared to baseline values in 15 horses (H1–H15).

Fifteen horses (designated H1–H15 and representing five breeds) had baseline TNF concentrations measured; nine horses were low (< 10,000 pg/mL), three horses were moderate (10,000–100,000 pg/mL), and three horses were high (> 100,000 pg/mL) (Table 1). Within one hour of IV LPS administration, the change in plasma concentrations of TNFα varied widely among horses; the percent change in plasma TNFα concentration ranged from -9% to 100% (Fig. 1, Table 1). Four horses (H4, H6, H8 and H9) were “high responders” (≥ 50% increase in plasma TNFα concentration), seven (H1, H2, H3, H5, H7, H10, H12, H14) had a minimal response (< 20% increase in plasma TNFα concentration), and three (H11, H13 and H15) had a moderate response (20–49%).

**Table 1:** Effect of IV LPS infusion on plasma TNFα concentrations in 15 horses.

Chr	EquCab3.0 Position	Ref	Alt	Consequence	Protein Position	SNP ID
25	22404058	G	T	Synonymous	260 L	rs782825011
25	22404206	G	A	Missense	D310N	rs782857623
25	22404251	A	C	Missense	K325Q	rs782902089
25	22404355	C	T	Synonymous	359 F	rs1144637357
25	22404541	C	T	Synonymous	421 G	rs1145524760
25	22404891	T	C	Missense	M538T	rs782839327
25	22405051	T	C	Synonymous	591 F	rs782871951
25	22405084	G	A	Synonymous	602 L	rs782895582
25	22405211	A	G	Missense	M645V	rs782844813

High, moderate, and low responders are defined as ≥ 50%, 20–49%, and < 20% increase in plasma TNFα concentration, respectively. Baseline categories indicate pre-LPS administration TNFα concentrations as high (> 100,000 pg/mL), moderate (10,000–100,000 pg/mL), and low (< 10,000 pg/mL).

When both baseline and response TNFα concentrations were categorized as high, moderate, or low, and subjected to a Kendall’s tau test for ordinal-ordinal data, the correlation was − 0.519, with *P* = 0.033, suggesting that a correlation does exist between baseline and response.

### Identification of TLR4 and MD2 variants, haplotype construction, and association analysis

Sequencing of the *TLR4* gene exons revealed nine different variants, all previously reported (https://www.ebi.ac.uk/eva/; Table 2)^27^, comprising four haplotypes among the 15 study horses (Supplementary Table S2). Four single nucleotide polymorphisms were missense changes, resulting in a predicted AA change; the remaining five variants were synonymous (no AA change). Of the four haplotypes, three shared an AA sequence, i.e., the haplotypes differed at the base pair level, but not at the AA level. The two predicted AA sequences are shown in Supplementary Figures S1 and S2.

**Table 2:** *TLR4* variants identified in 15 horses with variable responses to intravenous LPS infusion.

Horse, grouped by TNFα response (Breed)	Baseline TNFα (pg/mL) [Baseline category]	TNFα (pg/mL) one hour after LPS infusion	% increase in TNFα concentration
High responders			
H4 (Standardbred)	25.2 [Low]	310	1,129
H6 (Saddlebred)	115 [Low]	549	377
H8 (Thoroughbred)	322 [Low]	1089	238
H9 (Paint)	0 [Low]	93	N/A
Moderate responders			
H11 (Thoroughbred)	66,606 [Moderate]	86,792	30.3
H13 (Standardbred)	277 [Low]	415	50
H15 (Quarter Horse)	145 [Low]	264	82.1
Low responders			
H1 (Paint)	834,000 [High]	821,000	− 1.6
H2 (Quarter Horse)	0 [Low]	0	N/A
H3 (Paint)	155,147 [High]	156,362	0.80
H5 (Thoroughbred)	18,225 [Moderate]	20,520	12.6
H7 (Standardbred)	508 [Low]	560	10.2
H10 (Thoroughbred)	32,440 [Moderate]	29,760	− 8.3
H12 (Thoroughbred)	5,528 [Low]	5120	− 7.4
H14 (Quarter Horse)	199,959 [High]	207,868	4.0

Chr: equine chromosome. EquCab3.0 Position: base pair position using EquCab3.0 genome build. SNP ID: drawn from European Variation Archive. Note that “Ref” and “Alt” alleles from EquCab3.0 are occasionally flipped compared to the GenBank sequence (NP_001093239.2).

Three exonic *MD2* variants were identified among 11 horses compared to the GenBank reference sequence (Table 3); all three variants were previously reported (https://www.ebi.ac.uk/eva/)^27^. Only one of the *MD2* variants was missense; the other two were synonymous. Four haplotypes were observed for *MD2* in this horse population (Supplementary Table S3); the wild type sequence was most prevalent (accounting for of 18/22 total haplotypes). Of the four haplotypes, three shared an AA sequence. Predicted AA sequences are shown in Supplementary Figure S3 and S4.

**Table 3:** *MD2* variants identified in 11 horses with variable responses to LPS infusion.

Chr	EquCab3 Position	Ref	Alt	Consequence	Protein Position	SNP ID
9	12923460	G	A	Synonymous	55 K	rs3432833610
9	12923430	C	T	Synonymous	65 F	rs1146887989
9	12919464	G	C	Missense	R106T	rs1147734285

Chr: equine chromosome. EquCab3.0 Position: base pair position using EquCab3.0 genome build. SNP ID: drawn from European Variation Archive. Note that “Ref” and “Alt” alleles from EquCab3.0 are occasionally flipped compared to the GenBank sequence (NP_001075367.1).

When categorized by TNFα response to IV LPS infusion (i.e. high, moderate, or low response), there was no association between either *TLR4* or *MD2* haplotype and response (*P* value = 0.814 and 0.602, respectively). Additionally, no individual variants within *TLR4* or *MD2* were associated with response (*P* values all _ > 0.058). Baseline TNFα (when categorized as high, moderate, or low) was associated with *TLR4* haplotype (*P* value = 0.018), but not *MD2* haplotype (*P* value = 0.316). However, follow-up pairwise comparisons of baseline TNFα categories (high, moderate, low) between each pair of *TLR4* haplotype, via two-tailed Cochran Armitage tests, revealed no significant differences after applying a Bonferonni correction (Table 4). When testing associations between individual variants and all phenotypes, all results were not significant, except for the individual baseline TNFα concentration for the missense *MD2* variant, which was significantly associated with phenotype even following permutation (*P* value = 0.046). This significant result was driven by horse H1, which had very high baseline TNFα concentration and was the only horse with *MD2* haplotype #4 (the missense variant).

**Table 4:** Pairwise two-tailed Cochran Armitage test p-values for categorical baseline TNFα values (high, moderate, low) and *TLR4* haplotype.

*TLR4* Haplotype	1	2	3
2	0.0494		
3	0.0275	0.6001	
4	0.1596	0.0675	0.0533

Following Bonferonni correction, a significant *P* would be ≤ 0.0083.

### Structure of equine TLR4-MD2-LPS complex

**Figure 2:**
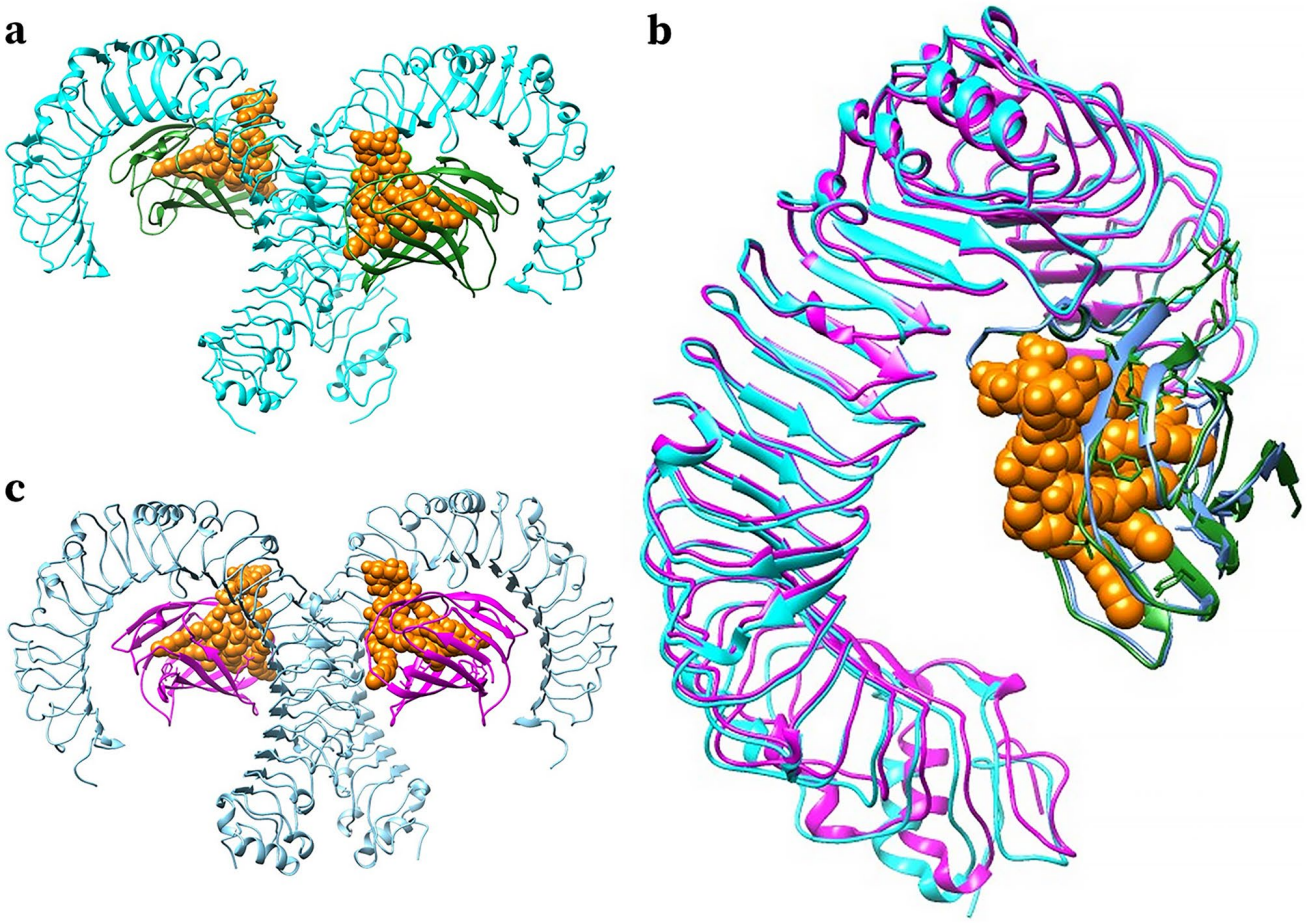
Construction of Equine TLR4-MD2-LP structure. (**a**) Structure of the human TLR4-MD2-LPS complex (PDB ID 3FXI)^19^, with TLR4 proteins (cyan blue), MD2 proteins (green), and LPS endotoxin (orange). (**b**) Merged equine (magenta) and human (cyan blue) TLR4 subunit, and merged equine (cornflower blue) and human (green) MD2 protein; LPS endotoxin (orange). (**c**) The complete structure of the equine TLR4-MD2-LPS complex, with TLR4 (sky blue), MD2 (magenta), and LPS endotoxin (orange).

The structure of equine TLR4-MD2-LPS was constructed to determine if the differing AA residues identified in study horses were close the LPS binding site. While the human TLR4-MD2-LPS structure is known (Fig. 2A; PDB ID 3FXI), the corresponding equine structures, as determined by haplotypes in the present study, have not been determined to date. Sequence homology between the human and equine proteins allowed the construction of an equine TLR4-MD2-LPS model. One unit of the equine TLR4-MD2-LPS structure was built using the human TLR4 and MD2 as templates, and the equine structure was then merged with the human structure (Fig. 2B). As expected, the merged structures showed similarities between the two species. Next, the complete model of equine TLR4-MD2-LPS was built (Fig. 2C).

**Figure 3:**
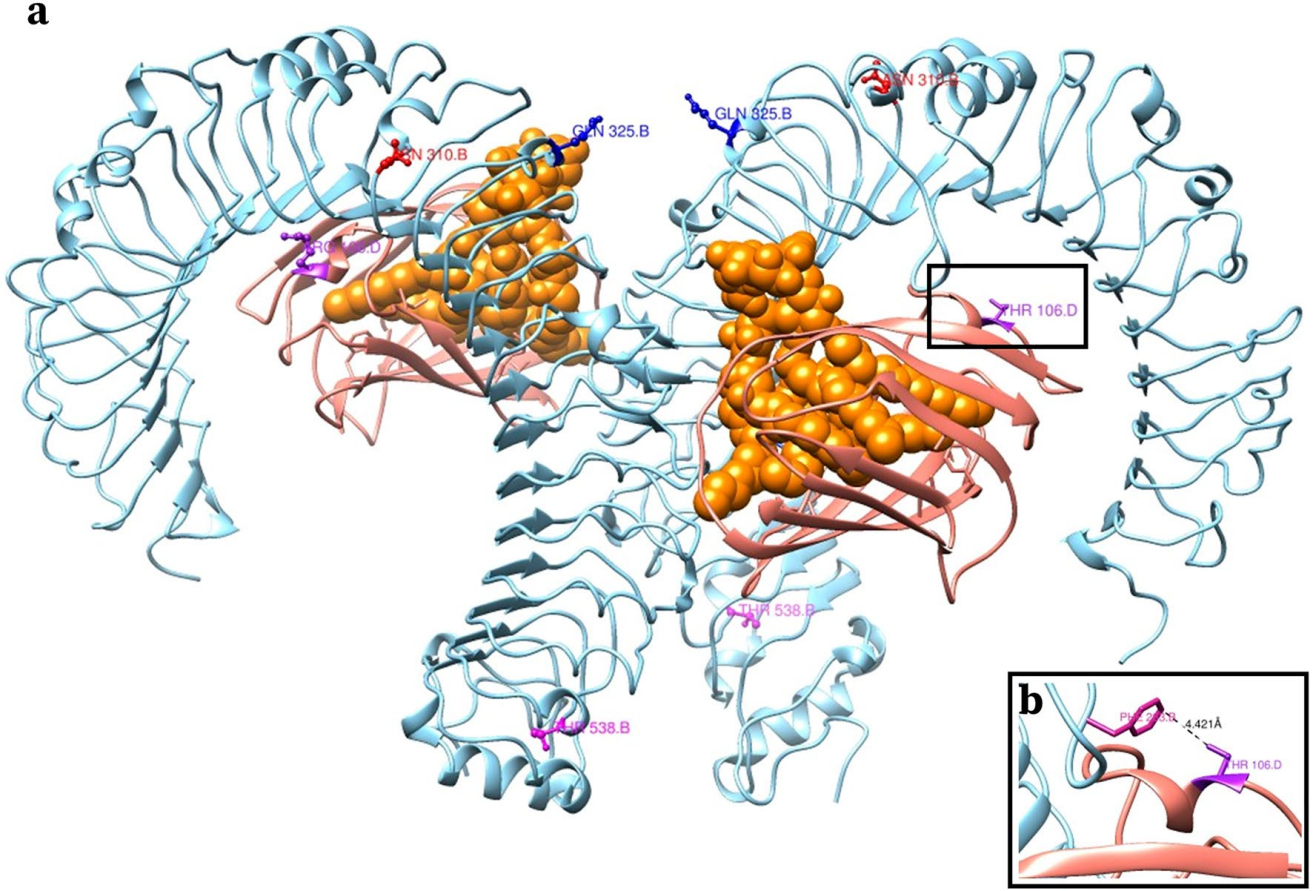
Complex 3: equine TLR4-MD2-LPS with TLRF-2:TLR4-2:MD2-1:MD2-2. a. The relevant amino acids are shown in a ball-and-stick model. b. The distance between MD2’s Thr106 and TLR4’s Phe263 (4.42 Å) is shown.

Haplotype analysis demonstrated that there were two TLR4 AA sequences and two MD2 AA sequences among the study horses. These AA sequences were designated TLR4-1 (Suppl Fig. S1), TLR4-2 (Supplementary Figure S2), MD2-1 (Supplementary Figure S3) and MD2-2 (Supplementary Figure S4). Each polypeptide combination (pair of haplotypes) observed among study horses were built (Fig. 3 and Supplementary Figures S5, S6, and S7). In TLR4-1, the relevant AA variations were Asp310, Lys325, and Met538. In TLR4-2, the relevant AA variations were Asn310, Gln325, and Thr538. MD2-1 had one AA variation, Arg106, whereas MD2-2 had Thr106. Four different TLR4-MD2-LPS complex formations were possible, given the haplotypes of the study horses: TLR4-1:TLR4-1:MD2-1:MD2-1 (designated as TLR4-MD2-LPS Complex 1), TLR4-2:TLR4-2:MD2-1:MD2-1 (designated as TLR4-MD2-LPS Complex 2), TLR4-2:TLR4-2:MD2-1:MD2-2 (designated as TLR4-MD2-LPS Complex 3), and TLR4-1:TLR4-2:MD2-1:MD2-1 (designated as TLR4-MD2-LPS Complex 4). These four structures were modeled to investigate possible effects of the variations on LPS binding.

The simulated model of Complex 1 (Supplementary Figure S5A) showed that: (1) the TLR4 Asp310 residues were not close to the LPS molecule, the TLR4-MD2 interface, or the dimer-dimer interface; (2) the TLR4 Lys325 residues were not close to the LPS molecule, and the distance between these two lysine residues from each subunit was 5.25 Å (Supplementary Figure S5B); (3) the TLR4 Met538 residues were located distant from relevant sites; (4) the MD2 Arg106 was physically close to AA in the neighboring TLR4 chain; specifically, two TLR4 AA (Asp209 and Phe263) were both within 3.0 Å (H-bonding distance) of MD2 Arg106 (Supplementary Figure S5C).

Complex 2 (Supplementary Figure S6A), just as in Complex 1, demonstrated that the TRL4 Asn310 residues were not near either the LPS molecule, the TLR4-MD2 interface, or the dimer-dimer interface. Also similar to Complex 1, the MD2 Arg106 residue was physically close to AA in the neighboring TLR4 chain. Complex 2 differed from Complex 1 in that the TLR4 Gln325 residues were 11.5 Å apart (Supplementary Figure S6B).

Complex 3 (Fig. 3) was observed in only one horse (H1), where its individual baseline TNFα concentration was significantly associated with the missense *MD2* variant (Arg106 versus Thr106; Fig. 3A). Complex 3 was identical to Complex 2 for TLR4 polypeptides, but *MD2* existed in this horse as two different haplotypes. One copy of MD2, included Arg106 that was stabilized by interactions with Asp209, as shown in Supplementary Figure S5C. However, the other copy of MD2 included Thr106, to which the closest TLR4 residue was 4.42 Å away (Fig. 3B).

Complex 4 (Supplementary Figure S7) exhibited matched MD2 chains, as in Complexes 1 and 2. Complex 4 had two different TLR4 polypeptides, varying at three AA residues (Supplementary Figure S7A). The Lys325 from one TLR4 chain was located 6.74 Å from the Gln325 on the other TLR4 chain (Supplementary Figure S7B).

Surface charges for each TLR4 and MD2 haplotype observed in the study population were also modeled (Supplementary Figure S8A–D). The varying 325 residue of TLR4 (Lys325 versus Gln 325) and the varying 106 residue of MD2 (Arg106 versus Thr106) demonstrate surface charge differences that may affect binding.

## Discussion

The low-dose IV LPS infusion model has been used extensively in equine research to study the pathophysiology of early gram negative septicemia and test the efficacy of potential drug candidates^8,9,13,28–34^. A strong inflammatory response to low-dose IV LPS is typically associated with clinical signs of systemic inflammation as well as increased TNFα serum concentrations by ≥ 50%^13,28,29^. However, up to 20% of horses fail to respond to this model^9^. One possible explanation is endotoxin tolerance, which is characterized by a diminished pro-inflammatory response to endotoxin that is recognized in animals and humans^35–37^. However, endotoxin tolerance typically subsides within 3 weeks of endotoxin exposure in horses^38^, making it an unlikely explanation in clinically normal horses. This study aimed to investigate TLR4 and MD2 variations among horses with different responses to LPS.

DNA sequencing of *TLR4* and *MD2* from horses representing multiple breeds with different TNFα dynamics after experimental IV LPS infusion revealed four haplotypes and two AA sequences for each gene. There were no associations between any haplotypes or single variant and TNFα response categories (high, moderate, low) for either gene. Similarly, when TNFα baseline concentrations were categorized as high, moderate, or low, there were no associations with haplotypes for either gene. The only significant association was between the individual baseline TNFα concentration and a missense *MD2* variant. However, this result was based on a single horse (H1) which had the highest baseline TNFα concentration and was the only horse to carry the *MD2* missense variant (the MD2-2 AA sequence). Baseline TNFα concentrations varied widely among study horses, in spite of meeting normal clinical inclusion criteria. This variability is discussed further below. H1 was a Paint, and while one other Paint horse (H3) also had a higher baseline TNFα concentration, the third Paint (H9) in the study had an undetectable baseline TNFα concentration; indeed, H9 had the lowest baseline value in the study. H1 may have been acutely exposed to gram negative bacteria prior to the LPS infusion in this study, accounting for the very high baseline TNFα concentration; however, this horse had no evidence of an ongoing inflammatory response and met the inclusion criteria as “normal” before the study began. Given the small sample size, conclusions regarding the significance of this single *MD2* missense variant association cannot be made. Previous in vitro*/ex-vivo* work evaluated responsiveness to LPS in a whole blood assay (n = 10 horses)^39^; the same four *TLR4* missense mutations were detected among these horses and, identical to the current findings, no relationship was observed between any variant and altered LPS response. Unlike the present study no *MD2* variants were reported from this previous small group of horses; however, their sample population consisted of only one breed (Dutch Warmblood)^39^.

Each haplotype combination was modeled as a 3D structure, using the known human TLR4 and MD2 structures as templates. This allowed visualization of each AA-changing variant’s position in relation to the LPS binding site, the TLR4-MD2 interface, and the dimer-dimer interfaces, with the aim of elucidating whether any of the AA changes might alter function and explain the variable TNFα values. There are six lipid chains in each LPS molecule, five of which are buried inside the hydrophobic pocket. The remaining chain is exposed to the surface of MD2 and forms hydrophobic interactions with TLR4^24^. Since LPS binds both TLR4 and MD2 proteins, variations in their critical LPS-binding domains or in overall protein structural integrity could lead to a significant effect. Complexes 1, 2, and 4 were not associated with any significant differences in TNFα values (baseline or response), although some of the AA changes could potentially result in alteration to attraction/repulsion at the quaternary protein structure level. For example, the variant Lys325 residues in Complex 1, which has two identical TLR4-1 subunits, are located just 5.25 Å apart. Lysine is a positively charged residue, and this small distance could create charge repulsion not observed in the wild type proteins. Similarly, also in Complex 1, the variant Arg106 residues in MD2-1 is close to TLR4’s Asp209, which likely creates a strong ionic attraction between the positively charged Arg106 and the negatively charged Asp209. Such small changes, though not statistically significant in the present work, may still contribute to overall protein stability.

Complex 3 contained one copy of the MD2-2 haplotype and therefore the *MD2* missense variant, which was the only variant significantly associated with TNFα baseline concentration. In the dimer-dimer complex, one MD2 protein had Arg106 and the other had Thr106. One monomer (consisting of one TLR4 subunit and one MD2 subunit) having Arg106 is predicted to be stabilized by this residue interacting with TLR4’s Asp209 (as shown in Supplementary Figure S5C), while the other monomer having Thr106 is predicted to have no substantial interactions with the neighboring TLR4 protein (Supplementary Figure S5B). Threonine, a polar, uncharged AA is smaller than arginine, which is a basic AA residue, and the closest TLR4 residue (Phe263) was 4.42 Å distant. It is unclear how this single missense change translates into the high baseline TNFα concentration in this single horse (H1). It is possible that the significant association is spurious and this missense change is well-tolerated. In fact, the European Variant Archive (https://www.ebi.ac.uk/eva/), under accession datasets PRJEB28306 and PRJEB9799, has whole genome sequence from 94 horses representing over two dozen breeds; nine of these horses are heterozygous for the same *MD2* missense variant (minor allele frequency = 0.049). This demonstrates that the variant is not overly rare among horses, although the impact of this variant on MD2 protein function, response to LPS, and resulting TNFα concentration response, are still unknown. Ultimately, it is important to remember that H1 was clinically normal at baseline for all other parameters, yet very high for baseline TNFα concentration; the present protein modeling work has unfortunately not provided any meaningful additional explanation for this finding. Previous work has demonstrated that the MD2 122 locus, positioned on the outer lip of the MD2 pocket^40^, varies between species (mouse, human, and horse), and that the R122E change in equine MD2 (arginine and glutamic acid, amino acids with opposite charge), can impair activation of equine TLR4^41^. Importantly, previous work examining TLR4 and MD2 sequence differences between four species (human, mouse, horse, and hamster), did not include the currently described MD2 106 variant^42^. More in-depth studies of the MD2 106 locus, ideally in an in vitro model, and potentially with additional agonist ligands, may identify different outcomes with the variant compared to wild type.

TLR4-MD2 heterodimer binding is mediated by a primary contact surface of TLR4 with both negatively charged and positively charged regions, which are complementary to the positive and negative charges on the surface of MD2, respectively^43,44^. Mutations in these primary interfaces can disrupt TLR4-MD2 binding. Previous reports have demonstrated different surface charges between species; for example, species differences were noted in TLRF surface charge distribution when comparing human to bovine^19^. Other work demonstrated differences in electrostatic potential based on MD2 surface charge between different species, including horse, and also suggested that TLR4 missense variants could change surface charge distribution in addition to other structural changes^18^. Therefore, surface charge differences likely influence ligand (LPS) binding. The surface charges for each TLR4 and MD2 subunit observed in the equine study population were modeled (Supplementary Figure S8), and differences were observed. The varying 325 residue of TLR4 (Lys325 versus Gln 325) does affect surface charge, which may affect the dimer-dimer stability at that location. Similarly, the varying MD2 106 residue (Arg106, a basic residue, versus Thr106, a neutral residue) also affects surface charge; because this residue is in close proximity to TLR4’s Asp209 residue (acidic), the ionic interactions at this location may affect stability of the entire complex. While neither of these loci appears to directly affect LPS binding, even TLR4 and MD2 residues that do not directly participate in ligand binding can determine the signaling outcome of a given ligand^45^.

Baseline TNFα concentrations varied widely among the horses in this study, despite their normal clinical examinations, CBCs, and biochemical profiles at the time of TNFα measurements. While a Kendall’s tau test comparing baseline TNFα concentrations with response TNFα concentrations (each categorized as high, moderate, or low) suggested a significant negative correlation (*P* = 0.033), the data set was very small. Further, all horses (n = 3) that had a high baseline TNFα concentration were categorized as low responders, and every horse (n = 4) that was categorized as a high responder started with a low baseline TNFα concentration. It is possible that subclinical inflammation was present in horses with “moderate” or “high” TNFα concentrations, but to date, large-scale screening studies of TNFα concentrations in healthy horses have not been performed, and there is a paucity in the literature of correlations between TNFα concentrations and clinical status in this species^46^. It is also possible that initial high TNFα concentrations might prevent a further measurable response, rendering the response automatically to the “low” TNFα response category. Unexpectedly, two horses (H10 and H12) had decreased TNFα concentrations one hour after LPS infusion, the meaning of which is uncertain. Until more is known, it seems reasonable to continue to screen horses for TNFα concentrations, but perhaps not to use this metric as either inclusion or exclusion criteria; it may be prudent, though, to exclude horses with very high baseline cytokine levels in future studies. Future work could also include more controlled evaluation of the *TLR4* and *MD2* variants via in vitro stimulation of whole blood or PBMCs with increasing LPS doses and regular measurement of TNFα.

The major limitation to this study is the small sample size, with only 15 and 11 horses sequenced for *TLR4* and *MD2*, respectively. While horses are an excellent clinical and research model for endotoxemia, they remain an expensive animal to house, decreasing the available sample size. Another limitation is the lack of measurement of other cytokines; TNFα concentrations may not be the best marker for LPS efficacy. In addition, the absence of generated sequences in the study horses for introns or untranslated regions of *TLR4* or *MD2* poses another limitation. Many non-coding variants can still impact gene and protein expression; these were not assessed in the present study. Furthermore, the present study did not sequence the equine TNFα gene, which has variants in its promoter/5’-UTR and throughout its structure (https://www.ebi.ac.uk/eva/ and https://www.ensembl.org)^47,48^. Neither did this work examine gene sequence from LPS binding protein or CD14 in the study horses; both of these proteins are also involved in LPS binding. Future work would benefit from incorporation of sequence variant analysis in these genes as well. Finally, the possibility must be considered that some of the in vivo response may be due to TLR2-mediated signals (in addition to TLR4), owing to signaling contaminants in the LPS preparation. This would obviously only affect TNFα response values and not baseline concentrations.

The overall goal of this study was to determine the relationship between gene variants and TNFα profile. Future work should examine a larger cohort of horses and investigate associations between numerous inflammatory cytokines (e.g. TNFα, IL1β, IL6, and IL8) and TLR4 and MD2 variation. It is also possible that variants in other related genes (e.g., LPS binding protein, CD14, and TNFα) or other genetic predispositions are involved. Comparison among different breeds of horse might also show varying TLR4-MD2 complexes that could affect differences in LPS binding. This work combines for the first time *TLR4* and *MD2* genotyping and protein structural predictions with equine TNFα phenotypes and informs future work to further elucidate these molecular relationships.

## Methods

### Equine samples

Blood samples from 15 horses used in a previous study^9^ were evaluated for TNFα concentrations at the time of the original study; samples from both baseline and one hour after low-dose LPS (*E. coli* (0111:B4), Sigma Aldrich) administration were tested. Although details of LPS administration in these horses can be found elsewhere^9^, endotoxemia was induced by IV infusion of approximately 30 ng/kg (15 μg) LPS in 500 mL 0.9% sodium chloride over a 30 min period. Baseline TNFα concentrations were categorized as low (< 10,000 pg/mL), moderate (10,000–100,000 pg/mL), and high (> 100,000 pg/mL) (Table 1). The change in plasma concentrations of TNFα was measured one hour after IV LPS administration and reported as a percent change. (Fig. 1, Table 1). Concentrations of TNFα were measured using a commercially available enzyme-linked immunosorbent assay (ELISA) kit validated for use in horses (Horse ELISA Kits, R&D Systems, Minneapolis, MN)^49^. The mean of duplicate samples was recorded. The TNFα concentration data was generated in the context of the original study^9^, but is only being published now, together with sequencing data.

### TLR4 and MD2 gene sequencing

Three mL of whole blood was collected once each from every horse still available from the original study (n = 15) for DNA sequencing of the *TLR4* gene (approved by Purdue University’s Institutional Animal Care and Use Committee, #1803001719)^9^. Blood from 11 of the same 15 horses was available for *MD2* gene sequencing; four horses had inadequate sample for the *MD2* sequencing. For the *TLR4* gene sequencing, genomic DNA was isolated using the Qiagen DNeasy Blood and Tissue Kit following manufacturer’s instructions. Equine-specific primers were designed using equine *TLR4* mRNA sequence (GenBank accession # NM_001099769.2) (Supplementary Table S1) and Primer3 software (https://primer3.ut.ee/);^50^ the 1800 bp region that corresponded to the known extracellular domain coding region of the human *TLR4* gene was targeted. PCR was performed using New England BioLabs NEBNext Ultra II Q5 Master Mix, with PCR conditions following manufacturer instructions. PCR products were purified (Qiagen PCR purification kit) and sequenced at the Purdue University Bindley Bioscience Genomics Core Facility using the “WideSeq” service which leverages the Illumina DNA prep library construction kit and an Illumina MiSeq 500 cycle reagent cassette^51^.

The equine *MD2* coding sequence (GenBank accession number NM_001081898) is 482 bp long. Due to *MD2* having many introns, the direct genomic PCR methodology was passed over in preference for RT-PCR of mRNA, which was isolated from fresh whole blood samples (Qiagen Qiamp RNA Blood Mini Kit). PCR primers were designed based on equine reference *MD2* sequence (Supplementary Table S1) using Primer3. PCR products were generated using Invitrogen Superscript III One-step RT-PCR with Platinum Taq. The DNA sequencing of *MD2* PCR products was performed as described above. Gene variations in *TLR4* and *MD2* were defined as deviations from established sequences published as GenBank accession numbers NM_001099769.2 and NM_001081898, respectively.

### Variant phasing and haplotype prediction

Identified genetic variants within *TLR4* and *MD2* were phased with SHAPEIT2 software using the default recombination rate^52^. Since this population was not composed of a singular breed, an effective population size of 341 was used; this is the average of the estimated effective population sizes across 36 breeds^53^. Haplotypes were assessed using Haploview software^54^.

### Statistical analysis

The number of horses chosen for the study was based on available (live) horses with measured TNFα responses to LPS administration in a previous study^9^. All methods were performed in accordance with the relevant institutional guidelines and regulations. A Kendall’s tau test (Kendall rank correlation coefficient) for ordinal-ordinal data was used to evaluate correlation between TNFα baseline and response, each as categorical data (high, moderate, low)^55^. Fisher’s exact tests were performed to identify any significant relationships between all *TLR4* or all *MD2* haplotypes and TNFα serum concentration, for both categorical response (high, moderate, low) and categorical baseline (high, moderate, low). Follow-up pairwise comparisons were made between individual haplotypes, with categorical baseline TNFα serum concentrations as phenotypes, using Cochran Armitage testing; a Bonferonni multiple-testing correction was applied to these tests. Finally, PLINK software^56^ was used to test associations between individual variants and phenotypes (including categorical response, categorical baseline, and individual TNFα serum concentration; the latter used the measured TNFα concentrations as phenotypes), with an adaptive permutation applied to correct for multiple testing.

### Construction of three dimensional (3D) structure of equine TLR4, MD2, and complexes

Amino acid sequences of TLR4 and MD2 were determined in SnapGene software (Insightful Science; snapgene.com). Equine TLR4 and equine MD2 AA sequences were aligned with human TLR4 and human MD2, respectively, using Clustal Omega (https://www.ebi.ac.uk/Tools/msa/clustalo/) to determine the identity and similarity between the two species^57^. Equine and human TLR4 were found to be 73% identical and 82% similar, while MD2 was 65% identical and 81% similar. Because of the homology between species, it was possible to build 3D structures of equine TLR4 and MD2 proteins using human proteins as templates (Swiss-Model PDB viewer, http://www.expasy.org/spdbv/)^58,59^. The Protein databank ID of the human TLR4-MD2-LPS structure used in the present study was 3FXI; a simulated equine TLR4-MD2-LPS complex model was created using 3FXI as a template. The human structure included AA from N-terminal 27 to C-terminal 631 for TLR4 and AA 19 to 158 for MD2. The corresponding horse structure included AA from N-terminal 23 to C-terminal 625 for TLR4 and from N-terminal 21 to C-terminal 155 for MD2. The 3D structures were visualized using the UCSF Chimera program (https://www.cgl.ucsf.edu/chimera/),^60^ a structural biology tool that generates 3D images based on AA sequence. Distances between AA residues of interest (e.g., missense variants resulting in AA changes compared to reference) were measured in Angstroms (Å) based on the 3D models in UCSF Chimera. Lastly, surface charges for each TLR4 and MD2 monomer as observed in the study population were modeled in UCSF Chimera.

## Supplementary Information

Supplementary Information.

## Data Availability

All data generated or analyzed during this study are included in this published article and its Supplementary files.
