# Supplementary material for: TLR4 and MD2 variation among horses with differential TNFα baseline concentrations and response to intravenous lipopolysaccharide infusion
Source: Sci Rep. 2023 Jan 27;13:1486. doi: 10.1038/s41598-023-27956-y (PMC9883502; doi:10.1038/s41598-023-27956-y)

Supplemental Table 1. Primers used to sequence equine *TLR4* and *MD2* genes.

TLR4-Forward	5'CAACCATCTCTCCACCTTGATATTGAC3'
TLR4-Reverse	5'GTTCTTTACCAATTCATTCCTC3'
MD2-Forward	5'CTTCCATATTTCCTGAACCTG3'
MD2-Reverse	5'CTAGGTTGGTGTAAGAGGGT3'

Supplemental Table 2. Phased *TLR4* haplotypes for each study horse. Columns indicate base pair position starting from the first nucleotide of the start codon. Horse identifiers are at left. Horses are organized numerically within TNFα response category (Table 1). Every horse has two rows, each representing a single DNA strand. Four total haplotypes were identified among these horses (numbered 1-4), and are indicated at right. Green shading indicates wild-type allele, and pink shading indicates a variant allele, compared to the GenBank sequence.

	780	928	973	1077	1263	1613	1773	1806	1933	Haplotype	AA Sequence Abbrev.
**H4**	G	G	A	C	C	T	T	G	A	1	TLR4-1
	G	G	A	C	C	T	T	G	A	1	TLR4-1
**H6**	T	A	C	T	C	C	C	A	G	3	TLR4-2
	T	A	C	C	T	C	C	A	G	4	TLR4-2
**H8**	T	A	C	C	C	C	C	A	G	2	TLR4-2
	G	G	A	C	C	T	T	G	A	1	TLR4-1
**H9**	T	A	C	C	T	C	C	A	G	4	TLR4-2
	G	G	A	C	C	T	T	G	A	1	TLR4-1
**H11**	G	G	A	C	C	T	T	G	A	1	TLR4-1
	G	G	A	C	C	T	T	G	A	1	TLR4-1
**H13**	T	A	C	C	T	C	C	A	G	4	TLR4-2
	T	A	C	C	C	C	C	A	G	2	TLR4-2
**H15**	T	A	C	C	C	C	C	A	G	2	TLR4-2
	G	G	A	C	C	T	T	G	A	1	TLR4-1
**H1**	T	A	C	T	C	C	C	A	G	3	TLR4-2
	T	A	C	C	C	C	C	A	G	2	TLR4-2
**H2**	T	A	C	C	C	C	C	A	G	2	TLR4-2
	G	G	A	C	C	T	T	G	A	1	TLR4-1
**H3**	T	A	C	T	C	C	C	A	G	3	TLR4-2
	T	A	C	C	C	C	C	A	G	2	TLR4-2
**H5**	T	A	C	C	C	C	C	A	G	2	TLR4-2
	G	G	A	C	C	T	T	G	A	1	TLR4-1
**H7**	T	A	C	C	T	C	C	A	G	4	TLR4-2
	G	G	A	C	C	T	T	G	A	1	TLR4-1
**H10**	G	G	A	C	C	T	T	G	A	1	TLR4-1
	G	G	A	C	C	T	T	G	A	1	TLR4-1
**H12**	G	G	A	C	C	T	T	G	A	1	TLR4-1

	G	G	A	C	C	T	T	G	A	**1**	**TLR4-1**
**H14**	T	A	C	C	C	C	C	A	G	**2**	**TLR4-2**
	T	A	C	C	C	C	C	A	G	**2**	**TLR4-2**

Supplemental Table 3: Phased *MD2* haplotypes for each study horse. Columns indicate base pair position starting from the first nucleotide of the start codon. Horse identifiers are at left. Horses are organized numerically within TNFα response category (Table 1). Every horse has two rows, each representing a single DNA strand. Four total haplotypes were identified among these horses (numbered 1-4), and are indicated at right. Green shading indicates wild-type allele, and pink shading indicates a variant allele, compared to the GenBank sequence. The wild type haplotype (haplotype #1) is the most prevalent.

	203	233	355	Haplotype	AA Sequence Abbrev
H6	G	C	G	1	MD2-1
	G	C	G	1	MD2-1
H9	A	C	G	2	MD2-1
	G	C	G	1	MD2-1
H11	G	C	G	1	MD2-1
	G	C	G	1	MD2-1
H13	G	C	G	1	MD2-1
	G	C	G	1	MD2-1
H15	A	C	G	2	MD2-1
	G	C	G	1	MD2-1
H1	G	C	C	4	MD2-2
	G	C	G	1	MD2-1
H3	G	C	G	1	MD2-1
	G	C	G	1	MD2-1
H5	G	C	G	1	MD2-1
	G	C	G	1	MD2-1
H7	G	C	G	1	MD2-1
	G	C	G	1	MD2-1
H10	G	T	G	3	MD2-1
	G	C	G	1	MD2-1
H12	G	C	G	1	MD2-1
	G	C	G	1	MD2-1

Suppl. Fig. 1: Predicted amino acid sequence for *TLR4* haplotype 1 (designated AA sequence TLR4-1). Variants are highlighted, with missense variants in red and synonymous variants in blue (compared to NCBI Reference Sequence NP_001093239.2). Brackets indicate sequence used in constructing polypeptide complexes.

MMPPTRLAGTLIPAMAFLSCLR[PESWDPCVQVVPNTTYQCMDLNLYKIPE 50
NIPTSTKELDLSFNPLKELGSHSFSNFPELQVLDLSRCEIEMIEDDAYQG 100
LNHLSTLILTGNPIRSLALGAFSGLSSLQTLVAVETKLSSLEKFPIGHLK 150
TLKELNVAHNLIHSFKLPEYFSKMPNLEHLDLSNNKIQNISHEDLRVLHQ 200
MPLLNLSLDLSLNPLEFIQPDAFKEIKLHKLTLRSNFDSIDVMKSCIQGL 250
AGLKVNRLV**L**GEFKNERKLERFDTSALRGLHNLTIEEFRLAYIDNYSSKD 300
SIDLLNCLA**D**ISKISLVSLDLGNL**K**DFPKGFGWQDFELVNCRIEGFPTLE 350
LTSLKRLV**F**TSNKDMKSFNEVKLPSLEFLDLSRNRLSFKSCCSEADLKTT 400
RLKHLDLSFNDVISMSSNFM**G**LEQLEHLDFQHSTLKQASDFPVFLSLKNL 450
RYLDISYTNTRVVFHGIFDGLVSLQVLKMAGNSFKDNFLPNIFREMTNLT 500
TLDLSKCNLEQVSQEAFCLLPRLRVLNMSHNNLLFLD**M**LPYKPLHSLQIL 550
DCSFNRIVAFKWQELQHFPSSLASLNLTQNDFACVCEYQS**F**LQWVKDQRQ 600
L**L**VEVEHLVCAIPLQMRGMPVLGFN]NATCQISKTIVGGSVFSIL**M**VSVIA 650
VLVYKFYFHLMLLAGCKKYGRGESIYDAFVIYSSQDEDWVRNELVKNLEE 700
GVPPFQLCLHYRDFIPGVAIAANIIQEGFHKSRKVIVVVSQHFIQSRWCI 750
FEYEIAQTWQFLSSRAGIIFIVLHKLEKSLLRQQVELYRLLNRNTYLEWE 800
DSVLGRHIFWRRLRKALLDGKPWSPAGTADAAESRQHDAETST 843

Suppl. Fig. 2: Predicted amino acid sequence for *TLR4* haplotypes 2, 3, and 4 (designated AA sequence TLR4-2). While these three haplotypes vary at the base pair level, they do not vary at the amino acid level. Variants are highlighted, with missense variants in red and synonymous variants in blue (compared to NCBI Reference Sequence NP_001093239.2). Brackets indicate sequence used in constructing polypeptide complexes.

MMPPTRLAGTLIPAMAFLSCLR[PESWDPCVQVVPNTTYQCMDLNLYKIPE 50
NIPTSTKELDLSFNPLKELGSHSFSNFPELQVLDLSRCEIEMIEDDAYQG 100
LNHLSTLILTGNPIRSLALGAFSGLSSLQTLVAVETKLSSLEKFPIGHLK 150
TLKELNVAHNLIHSFKLPEYFSKMPNLEHLDLSNNKIQNISHEDLRVLHQ 200
MPLLNLSLDLSLNPLEFIQPDAFKEIKLHKLTLRSNFDSIDVMKSCIQGL 250
AGLKVNRLV**L**GEFKNERKLERFDTSALRGLHNLTIEEFRLAYIDNYSSKD 300
SIDLLNCLA**N**ISKISLVSLDLGNL**Q**DFPKGFGWQDFELVNCRIEGFPTLE 350
LTSLKRLV**F**TSNKDMKSFNEVKLPSLEFLDLSRNRLSFKSCCSEADLKTT 400
RLKHLDLSFNDVISMSSNFM**G**LEQLEHLDFQHSTLKQASDFPVFLSLKNL 450
RYLDISYTNTRVVFHGIFDGLVSLQVLKMAGNSFKDNFLPNIFREMTNLT 500
TLDLSKCNLEQVSQEAFCLLPRLRVLNMSHNNLLFLD**T**LPYKPLHSLQIL 550
DCSFNRIVAFKWQELQHFPSSLASLNLTQNDFACVCEYQS**F**LQWVKDQRQ 600
L**L**VEVEHLVCAIPLQMRGMPVLGFN]NATCQISKTIVGGSVFSIL**V**VSVIA 650
VLVYKFYFHLMLLAGCKKYGRGESIYDAFVIYSSQDEDWVRNELVKNLEE 700
GVPPFQLCLHYRDFIPGVAIAANIIQEGFHKSRKVIVVVSQHFIQSRWCI 750
FEYEIAQTWQFLSSRAGIIFIVLHKLEKSLLRQQVELYRLLNRNTYLEWE 800
DSVLGRHIFWRRLRKALLDGKPWSPAGTADAAESRQHDAETST 843

Suppl. Fig. 3: Predicted amino acid sequence for *MD2* haplotypes 1, 2, and 3 (designated AA sequence MD2-1). While these three haplotypes vary at the base pair level, they do not vary at the amino acid level. Variants are highlighted, with the missense variant in red and synonymous variants in blue (compared to NCBI Reference Sequence NP_001075367.1). Brackets indicate sequence used in constructing polypeptide complexes.

```
MFSFMLFFTLFSSIFPEPEE[LRWICNSSDMSIWYTYCDNMKSPISINLEP  50
CIELKGTRGHLHMLFVPRRDIKKLYFNLYLTMNSLEFPMRKEVICRGSDD  100
DYSFCRALKGETVNTTVSFSFRGMRFPKGRYSCIAEAVVGNTEEALFCLN  150
FTLLH]QPSFN                                        160
```

Suppl. Fig. 4: Predicted amino acid sequence for *MD2* haplotype 4 (designated AA sequence MD2-2). Variants are highlighted, with the missense variant in red and synonymous variants in blue (compared to NCBI Reference Sequence NP_001075367.1). Brackets indicate sequence used in constructing polypeptide complexes.

MFSFMLFFTLFSSIFPEPEE[LRWICNSSDMSIWYTYCDNMKSPISINLEP 50
CIEL**K**GTRGHLHML**F**VPRRDIKKLYFNLYLTMNSLEFPMRKEVICRGSDD 100
DYSFC**T**ALKGETVNTTVSFSFRGMRFPKGRYSCIAEAVVGNTEEALFCLN 150
FTLLH]QPSFN 160

Suppl. Fig. 5. Complex 1: equine TLR4-MD2-LPS with TLR4-1:TLR4-1:MD2-1:MD2-1. a. The relevant amino acids are shown in ball and stick model. b. The distance between two TLR4 Lys325 residues (5.25A). c. The distance between MD2's Arg106 and TLR4's Asp209 (2.67A). TLR4's Phe263 is also within 3.0A.

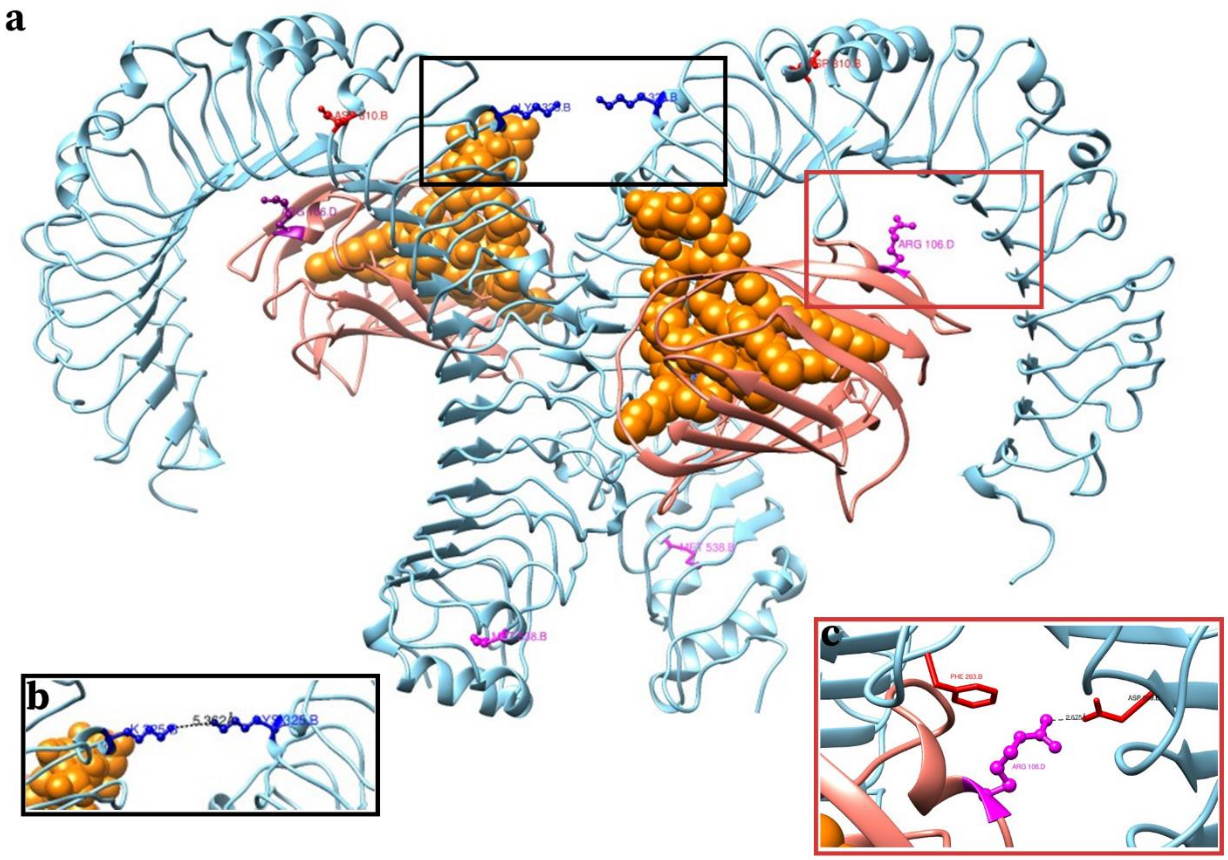

Suppl. Fig. 6. Complex 2: equine TLR4-MD2-LPS with TLR4-2:TLR4-2:MD2-1:MD2-1. a. The relevant amino acids are shown in ball and stick model. b. The distance between two Gln325 residues (11.5A).

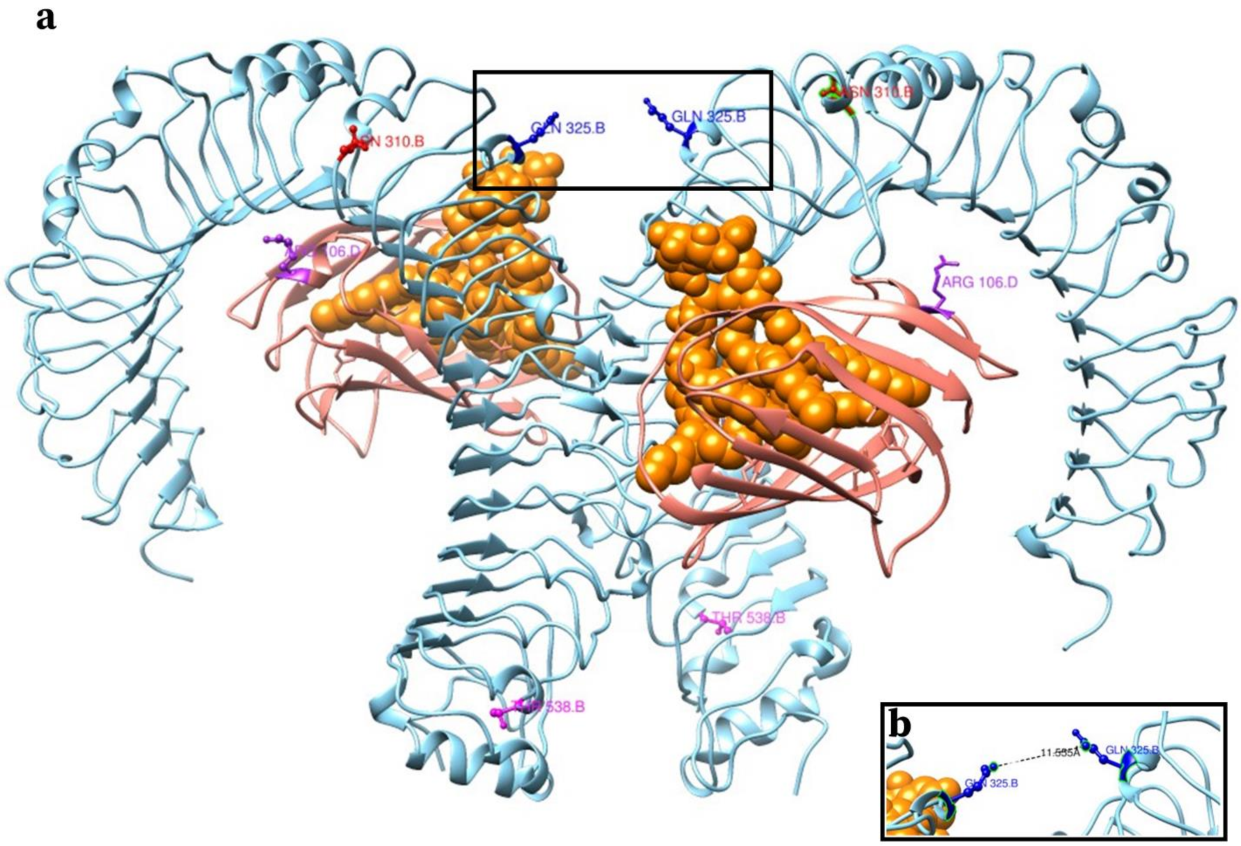

Suppl. Fig. 7. Complex 4: equine TLR4-MD2-LPS with TLR4-1:TLR4-2:MD2-1:MD2-1. a. The relevant amino acids are shown in ball and stick model. b. The distance between two Lys325 and Gln325 residues (6.74A).

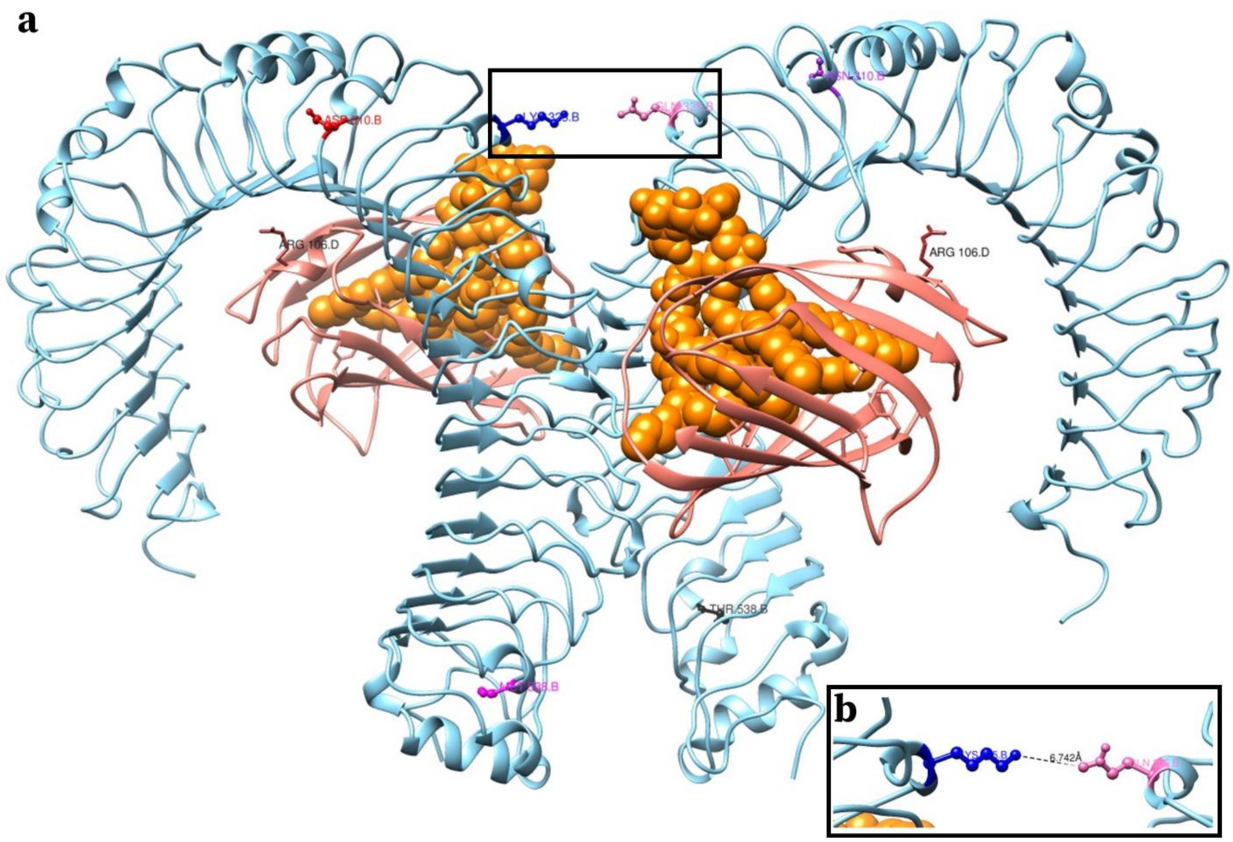

Suppl. Fig 8. Surface charges of TLR4 and MD2 proteins with variants observed among study horses. UCSF chimera was used to build each protein model. Potential values were set to -10 and +10 for negative and positive charges, respectively. Contour values were set to -5 and +5. Blue color = positive charge; red color = negative charge; white color = neutral charge. a. TLR4-1, demonstrating the Lys325 residue. b. TLR4-2, demonstrating the Gln325 residue. c. MD2-1, demonstrating the Arg106 residue. d. MD2-2, demonstrating the Thr106 residue.

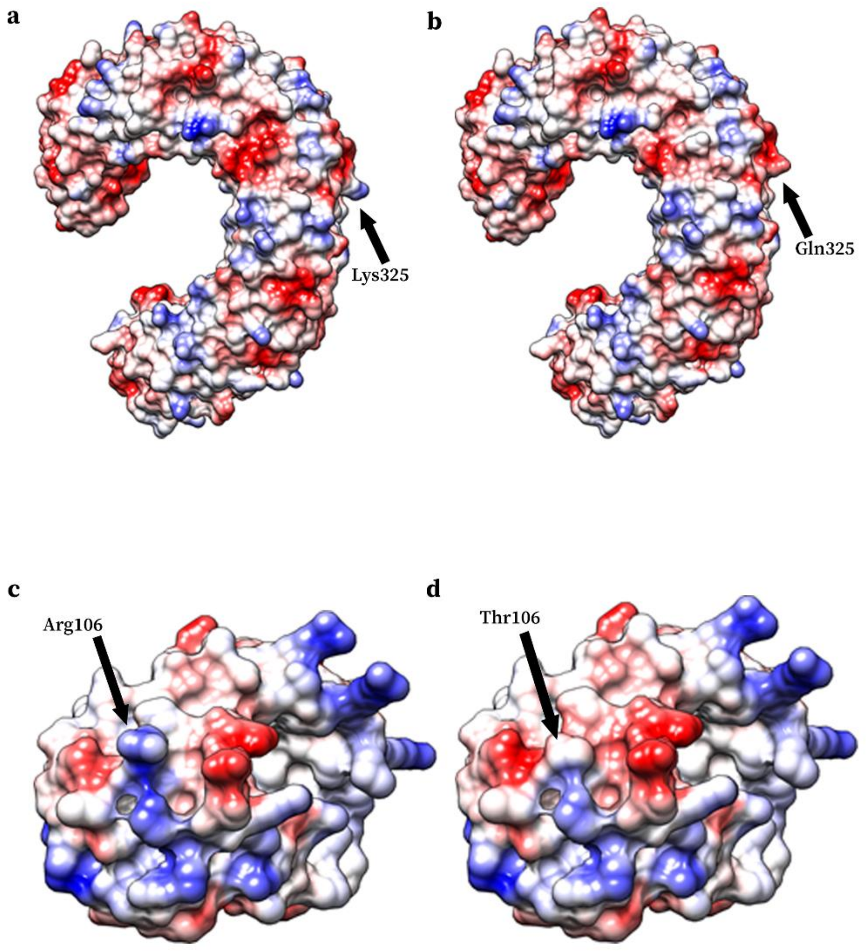

Supplement: Supplementary file 1 — Supplementary Information. [file 41598_2023_27956_MOESM1_ESM.pdf]
